# Segmentation-Guided Preprocessing Improves Deep Learning Diagnostic Accuracy and Confidence of Ameloblastoma and Odontogenic Keratocyst in Cone Beam CT Images—A Preliminary Study

**DOI:** 10.3390/diagnostics16030416

**Published:** 2026-02-01

**Authors:** Xinyue Zhang, Yuxuan Yang, Chen Zhong, Jupeng Li, Gang Li

**Affiliations:** 1 Department of Oral and Maxillofacial Radiology, Peking University School and Hospital of Stomatology & National Center of Stomatology & National Clinical Research Center for Oral Diseases & National Engineering Research Center of Oral Biomaterials and Digital Medical Devices, No. 22, Zhongguancun South Avenue, Haidian District, Beijing 100081, China; 1810303109@pku.edu.cn; 2 School of Electronic and Information Engineering, Beijing Jiaotong University, Beijing 100044, China; 23125011@bjtu.edu.cn (Y.Y.); 23120170@bjtu.edu.cn (C.Z.); lijupeng@bjtu.edu.cn (J.L.)

**Keywords:** medical image preprocessing, segmentation, explainable AI, cone-beam CT, ameloblastoma, odontogenic keratocyst

## Abstract

**Objectives**: The differential diagnosis of ameloblastoma and odontogenic keratocyst is essential for surgical planning and patient counseling. While deep learning (DL)-based methods show promising potential in this domain, their clinical translation remains challenging due to insufficient interpretability. This study aims to introduce segmentation-guided preprocessing approaches to provide support for the clinical implementation of computer-aided diagnosis systems. **Methods**: This study evaluated the performance of an InceptionV3 model on 128 pathologically confirmed CBCT scans (AME: 64; OKC: 64) by 5-fold cross-validation. Four experimental inputs were compared: (1) Original slice; (2) Bounding-box ROI; (3) Precise segmentation ROI; and (4) Moderately expanded ROI. All models were trained under the same settings. Assessment was conducted on both the slice and patient levels, incorporating accuracy, recall, precision, F1-score, and the area under the receiver operating characteristic curve (AUC). Grad-CAM visualization and confidence curve analysis were employed to verify models’ attention patterns and diagnostic confidence. **Results**: All models based on segmentation-guided ROI significantly outperformed models based on original slice. The moderately expanded ROI achieved optimal performance. The bounding-box ROI provided competitive performance with higher recall. Grad-CAM confirmed improved attention localization, while confidence curve analysis showed more consistent and reliable prediction patterns across slices. **Conclusions**: Segmentation-guided preprocessing represents an effective and clinically relevant approach for jaw lesion diagnosis and enhances interpretability.

## 1. Introduction

Deep learning (DL) is making significant strides in oral and maxillofacial radiology, offering promising potential for the diagnosis of jaw cystic lesions—pathologies that often appear as radiolucent areas. Recent research has focused on classifying these lesions using various imaging modalities [1,2], with early studies primarily concentrating on 2D panoramic radiographs [3,4,5]. However, 2D images are prone to anatomical superimposition and distortion, which can limit diagnostic accuracy. As a result, there is a shift toward utilizing 3D imaging techniques, particularly cone beam computed tomography (CBCT), for enhanced visualization of multilocularity and bone expansion patterns in jaw lesions. A comparative study by Lee et al. demonstrated superior diagnostic performance with a CBCT-based model over a panoramic-based model [6].

A single CBCT scan generates hundreds of slices used to reconstruct the jaw and associated lesions. However, not all of these slices are relevant for training deep learning models, making slice selection a key challenge. Manual selection of representative slices relies heavily on expert knowledge and is labor intensive [6,7]. On the other hand, mechanical sampling methods, such as selecting every third slice, can introduce non-informative noise from margin regions, which may degrade model performance [8]. Additionally, optimal preprocessing of medical imaging data for AI models remains an ongoing area of exploration [9].

Previous studies have commonly applied intensity- and frequency-based enhancement techniques—such as inverse logarithmic transformation, histogram equalization, and Fourier transformation—to improve feature visibility [3,5,10,11]. In contrast, recent studies focus on Region of Interest (ROI) processing. In this area, manual rectangular cropping has been used to reduce background interference when classifying jaw lesions [5,6,7,8]. More recently, Panyarak et al. [12] introduced the Extended Cropping and Padding (ECAP) technique, which aims to preserve contextual information around jaw lesions.

Simultaneously, object detection networks like EfficientDet-D4 [13], YOLO [11,14,15], and Detect-Net [16,17,18] have been leveraged to automatically extract bounding-box ROIs, effectively combining localization and classification tasks. These bounding-box ROIs typically encompass both the lesion and surrounding tissue, meaning the model processes a composite region. To address this, recent morphology-aware approaches—such as manually tracing lesion borders or using segmentation-assisted dual-branch classification networks—have been introduced to provide more precise lesion delineation [19,20].

Inspired by these studies, we developed a segmentation-guided ROI extraction approach to ensure that only informative regions are fed into the model, thus enhancing the recognition of complex pathological patterns. Clinically, this design is motivated by replicating the radiologist’s diagnostic process; first by identifying the lesion, then zooming in to assess its shape, internal structure, and the surrounding bone interface. However, it remains unclear whether a bounding-box ROI, a precise segmentation focusing strictly on the lesion’s shape and internal structure, or a moderately expanded ROI (which includes the surrounding bone interface) is the most effective approach.

To explore this further, we conducted a proof-of-concept study to compare the performance of the three aforementioned ROI types against the original slice in differentiating ameloblastoma (AME) from odontogenic keratocyst (OKC) on CBCT axial images. In addition to evaluating performance, we employed explainable AI (XAI) techniques, such as Grad-CAM and confidence curve analysis, to interpret the model’s focus and diagnostic confidence. Our goal is to provide valuable insights that can guide future research and contribute to the standardization of model input strategies in this field.

## 2. Materials and Methods

### 2.1. Ethical Standards

The study protocol adhered to the principles outlined in the Declaration of Helsinki and received approval from the Ethics Committee of Peking University School and Hospital of Stomatology (IBR: PKUSSIRB-202495005). The research protocol complied with the Checklist for Artificial Intelligence in Dental Research guidelines.

### 2.2. Data Collection

A power analysis was performed to ensure adequate statistical power. The target effect size was conservatively defined as a 17.7% absolute improvement in accuracy, based on prior work [8]. In that study, the lower bound of the 95% confidence interval for model accuracy (76.2%) exceeded the clinically relevant baseline accuracy (58.5%) by 17.7%. To detect an effect of this magnitude with a two-sided α = 0.05 and 80% power, a minimum of 36 cases per group (72 total) was required. The final cohort of 128 patients therefore exceeded the required sample size.

The inclusion criteria comprised patients who underwent CBCT examination at our institution between January 2020 and December 2024 and had a confirmed histopathological diagnosis of either ameloblastoma (AME) or odontogenic keratocyst (OKC). Only scans of sufficient diagnostic quality, as determined by a board-certified oral and maxillofacial (OMF) radiologist, were included. The exclusion criteria were as follows: (1) age below 7 years; (2) prior history of surgery involving the lesion; and (3) nevoid basal cell carcinoma syndrome.

All scans were acquired from three CBCT devices—two NewTom scanners (Cefla S.C., Imola, Italy) and one i-CAT FLX scanner (Imaging Sciences International Inc., Hatfield, PA, USA). Imaging parameters were as follows: tube voltage of 110 kV; tube current of 1.0–11.0 mA; 360° rotation; scan time of 17.6 s; FOV of 12 cm × 8 cm, 16 cm × 16 cm for NewTom scanners; and tube voltage of 120 kV; tube current of 5 mA; 360° rotation, scan time of 8.9 s; FOV of 16 cm × 11 cm, 16 cm × 13 cm for i-CAT FLX scanner. The voxel size was 0.2–0.3 mm^3^.

### 2.3. Data Set Construction

**Figure 1:**
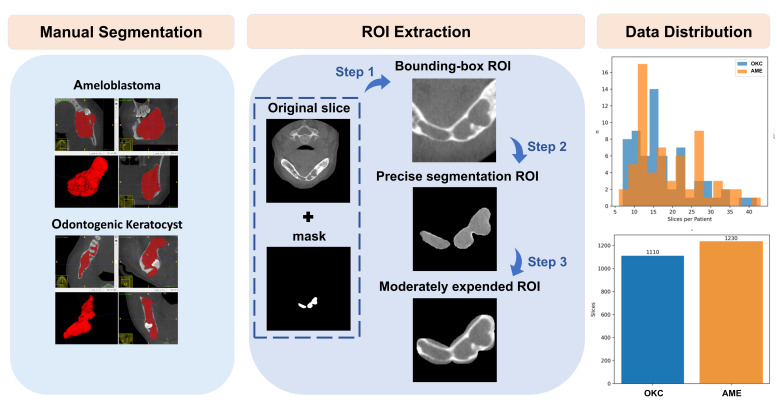
Workflow of segmentation-guided ROI preprocessing for dataset construction. The red area indicates jaw lesion. Step 1: Crop a mask-centered-square (expand 20%); Step 2: Zero out the non-mask area’s intensity; Step 3: apply morphological dilation.

CBCT scans were saved as DICOM format and subsequently imported into ITK-SNAP (v.3.8.0) for NIfTI format. An oral radiologist with 3 years of experience conducted manual segmentation under supervision of a board-certified OMF radiologist. Inter-observer reliability analysis of 16 cases (AME:OKC = 1:1) yielded an ICC of 0.999, indicating excellent agreement. Visualization of the volume measurements consistency is provided in Supplementary Figure S1. Only axial slices with a lesion greater than 50% of the maximum area were selected. After this, we adopted a strategic sampling approach by selecting every 5th slice to balance model performance with computational resources following the methodology described by Altun et al. [21] despite of the original axial slices available at 0.2–0.3 mm intervals. This process generated 2340 axial slices and their corresponding ROI images. The workflow of segmentation-guided ROI preprocessing is illustrated in Figure 1 and automated by Python (v.3.6.1).

The dataset was partitioned at the patient level to prevent data leakage so that slices from the same patient did not appear in both the training and testing sets. The detailed distribution of patients and slices across all folds is provided in Supplementary Table S1.

### 2.4. Model Development

#### 2.4.1. Model Architecture and Strategy

Following previous work [6,7,8], a pre-trained InceptionV3 model was fine-tuned for our binary classification task. The model was trained using AdamW optimizer with a two-phase schedule consisting of an initial warm-up phase for stabilization, followed by a cosine decay for fine-tuning. To mitigate overfitting, batch normalization, ReLU activation, and multiple dropout layers ranging from 0.6 to 0.3 were adopted. Detailed hyperparameters and the computational environment are provided in Supplementary Table S2.

The data were automatically divided into five groups for a 5-fold cross-validation (Table S3). The data augmentation pipeline for training included resizing, random horizontal flipping (50% probability), random rotation (±15°), and affine transformations with translation (±5%) and scaling (95–105%). For validation, only resizing was applied. All images were normalized to the range [0, 1]. We applied this augmentation strategy consistently across all experimental groups to ensure fair comparison.

#### 2.4.2. Mode-Adaptive Patient-Centric Sampling (MAPS)

The MAPS strategy employed distinct sampling modes for training and evaluation.

For training, we applied a balanced sampling strategy based on a weighted random sampling approach, a well-established method to handle data imbalance [22]. This method prevents the model from becoming biased toward patients contributing a larger number of images (Figure 1). Sampling weight is calculated on the relationship between image count per patient and the average image count.

1. Compute the average image count: $\bar{N}=\frac{N_{total}}{P}$
2. Calculate a patient-specific ratio: $r_{p}=\frac{N_{p}}{\bar{N}}$
3. Obtain the final weight: $W_{p}=\min\left(r_{p},2.0\right)$

Where $N_{\mathrm{total}}$ is the total image count, P is the total patient count, and $N_{p}$ is the image count for patient p.

For evaluation, a uniform sampling strategy was applied, ensuring that each patient had an equal probability of being selected in a batch. Once a patient was sampled, all corresponding images were included in the evaluation. This approach guarantees that each case contributes equally to the performance metrics, thereby providing a clinically realistic assessment of model performance.

### 2.5. Model Evaluation and Statistical Analysis

Quantitative evaluation was performed at both the slice and patient levels using the following metrics: accuracy, precision, recall, F1-score, receiver operating characteristic (ROC) curve, and area under the curve (AUC). Patient-level classification was derived using a soft-voting ensemble strategy based on probability averaging across slices. Model predictions were summarized using a confusion matrix to quantify true positives (TP), true negatives (TN), false positives (FP), and false negatives (FN) (Table 1). The formulas used to compute each metric are provided below.

- Confusion Matrix
- Accuracy provides the percentage of successful classifications: $\frac{TP+TN}{TP+TN+FP+FN}$
- Precision provides the percentage of true positive among all positive ratings: $\frac{TP}{TP+FP}$
- Recall provides the percentage of true positive among all true ratings: $\frac{TP}{TP+FN}$
- Specificity provides the percentage of true negative among all negative ratings: $\frac{TN}{TN+FN}$
- F1-score is calculated as the harmonic mean between recall and precision:
  $$2\times \frac{Precision\times Recall}{Precision+Recall}$$

Receiver operating characteristic (ROC) curve and areas under the curve (AUC): the ROC curve graphically illustrates the trade-off between recall (sensitivity) and 1 − specificity across all classification thresholds. The AUC quantifies overall discriminatory performance and ranges from 0 to 1, with higher values indicating better classification. DeLong’s test was used to assess the statistical significance of differences between AUC values.

To investigate how the different segmentation-guided ROI preprocessing methods alter the model’s decision-making mechanism, Grad-CAM was applied into the final mixed layer of InceptionV3 architecture. This technique generates visual heatmaps that highlight the image regions (typically in red) which contributed most to the model’s prediction [20,23].

Evaluating AI for clinical use requires moving beyond aggregate performance metrics like AUC. Understanding the specific failure modes of a model is critical [24]. The deep learning model outputs a continuous probability value via the Softmax layer, representing the likelihood that an input image belongs to a specific lesion category (AME = 1, OKC = 0). Confidence curves were plotted to visualize the model’s predicted probability for each image in sequential order. This visualization directly illustrates the internal consistency of predictions within individual cases.

## 3. Results

### 3.1. Demographic and Radiographic Characteristics

The demographic and radiographic characteristics of AME and OKC are summarized in Table 2. *p* values were calculated using the Mann–Whitney U test for age and chi-square test for categorical variables. No statistically significant differences were found between the AME and OKC groups with respect to age, sex, lesion location, or CBCT scanners (all *p* > 0.05). In contrast, significant differences were identified in locularity, cortical integrity, and root resorption between the two groups (all *p* < 0.01). These findings indicate that the AME and OKC cohorts are demographically comparable, while their distinct radiographic characteristics provide a clear and objective basis for the model to learn discriminative features.

### 3.2. Training Process

**Figure 2:**
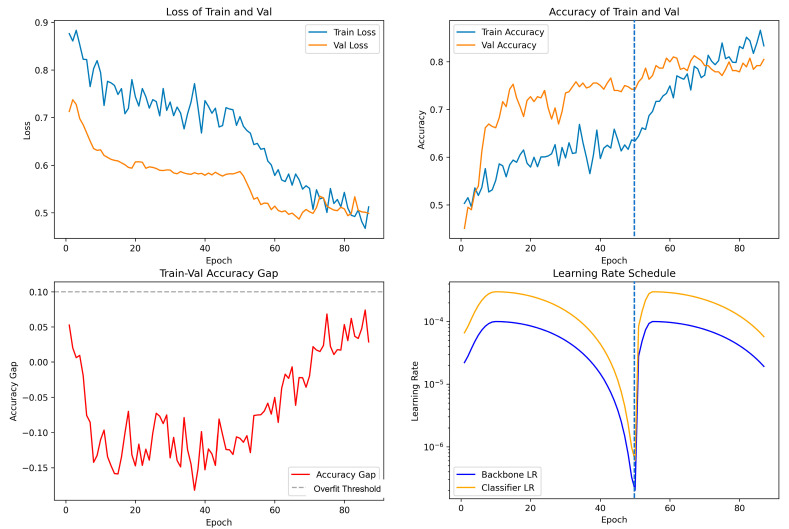
Training process of a representative fold from a 5-fold cross-validation in the moderately expanded ROI experiment. The training and validation loss curves (**upper left**), the training and validation accuracy curves (**upper right**); the train-val accuracy gap remains below the 0.1 threshold indicating by a dashed horizontal line (**lower left**), the dual-phase learning rate schedule, with a reset at epoch 51 (the blue dashed line), is displayed (**lower right**).

Figure 2 illustrates the training process of a representative fold from a 5-fold cross-validation in the moderately expanded ROI experiment. The convergent loss curves indicate a successful learning process. The initially higher validation accuracy suggests effective regularization. While the training accuracy typically converges with and slightly exceeded the validation accuracy, their gap remained below the 0.1 threshold, indicating no significant overfitting. The two-phase learning rate schedule proved effective; resetting learning rate at epoch 51 prompted a sustained performance increase, boosting the validation accuracy by approximately 7% and helping the model escape a potential plateau. Training was efficiently concluded via early stopping at epoch 87 when the validation performance stabilized.

### 3.3. Classification Performance

**Figure 3:**
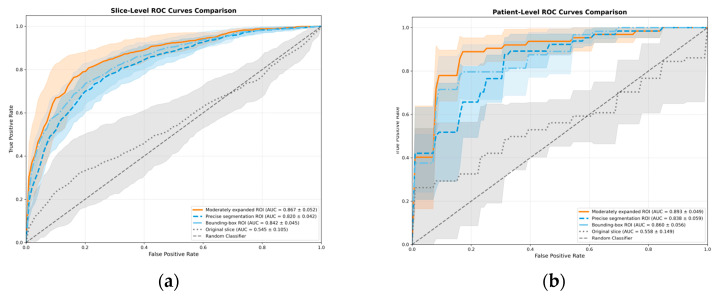
ROC curves of slice-level (**a**) and patient-level (**b**) for models trained on different ROI types, moderately expanded ROI, bounding-box ROI, precise segmentation ROI and original slice on the task of differentiating AME and OKC using CBCT axial images. The shaded area represents the range of the ROC curves across the 5-fold cross-validation.

**Figure 4:**
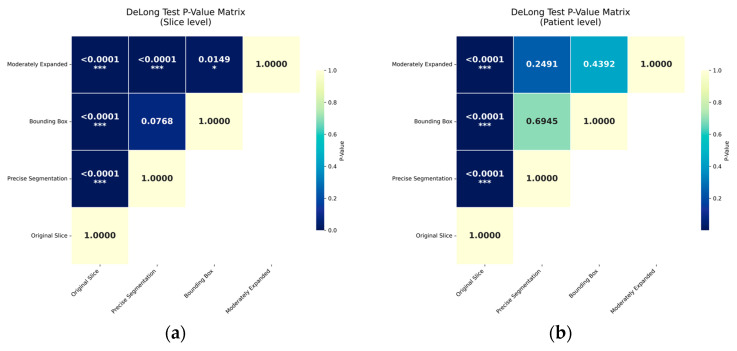
DeLong’s Test *p*-value Matrix at the slice-level (**a**) and patient-level (**b**). Statistical significance is indicated as * *p* < 0.05 and *** *p* < 0.001.

All segmentation-guided ROI extraction methods significantly outperformed the original slice method in AUC at both slice level and patient level (*p* < 0.0001) with reduced variance. At the slice level, the moderately expanded ROI achieved a significantly higher AUC than both the precise segmentation ROI (*p* < 0.0001) and the bounding-box ROI (*p* = 0.0149). A marginal, non-significant trend favored the bounding-box ROI over the precise segmentation ROI (*p* = 0.0768). This performance ranking was consistent at the patient level; however, the differences between the segmentation-guided methods did not reach statistical significance (*p* > 0.1) (Figure 3 and Figure 4). This lack of significance is further elucidated by the distributional overlap observed in their 95% Confidence Intervals (CIs), as detailed in Table S4.

The comprehensive quantitative results are summarized at both the slice and patient levels in Table 3 and Table 4. Radar charts in Figure S2 provides a comparative view of the model performance across groups.

At the slice level, the model utilizing moderately expanded ROI achieved the highest accuracy of 0.7727 ± 0.0483 and highest precision of 0.8351 ± 0.0896. The bounding-box ROI method also demonstrated strong performance, notably achieving the highest recall of 0.7955 ± 0.0947 and F1 score of 0.7603 ± 0.0761. The precise segmentation ROI method yielded comparatively lower results. At the patient level, the moderately expanded ROI approach again yielded the best overall results, attaining an accuracy of 0.8123 ± 0.0385, a precision of 0.8649 ± 0.0950 and an F1 score of 0.8009 ± 0.1101.

### 3.4. Interpretability Analysis

**Figure 5:**
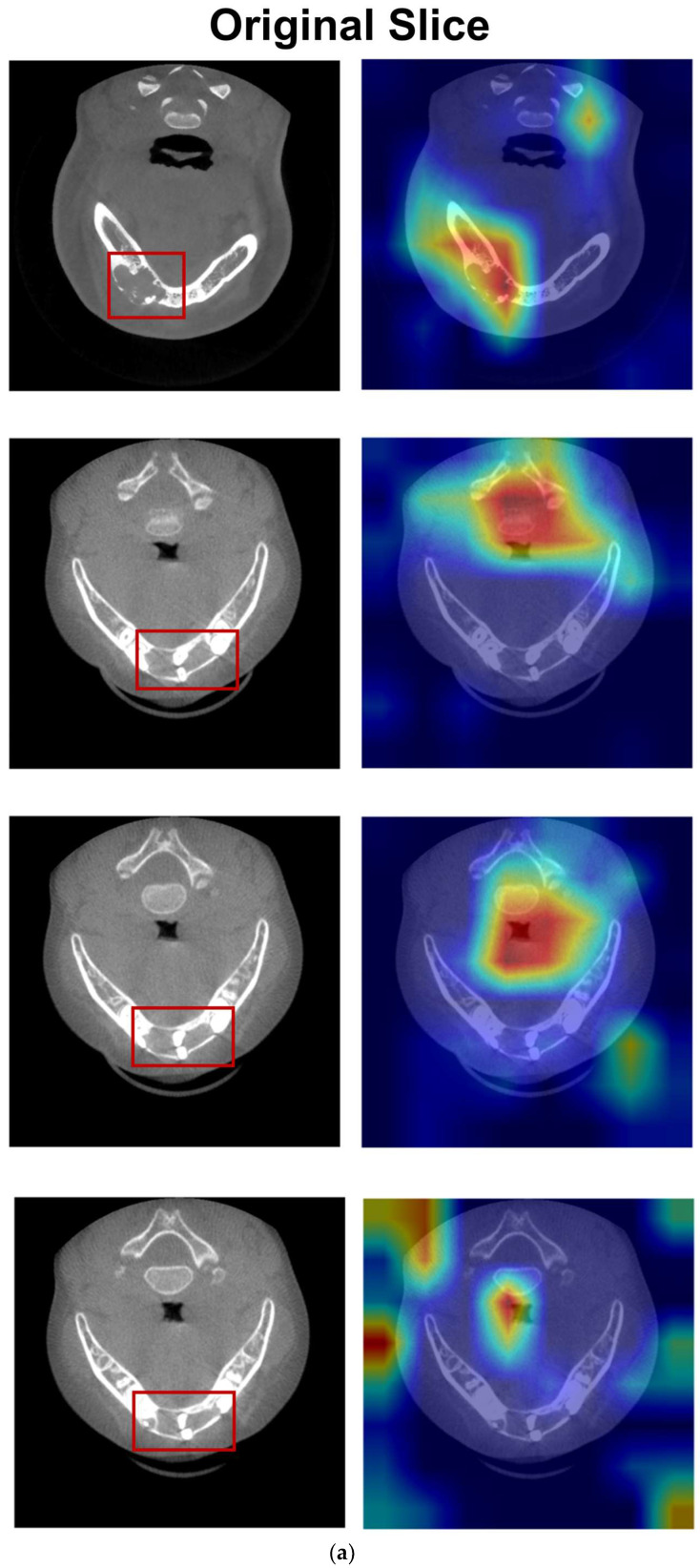

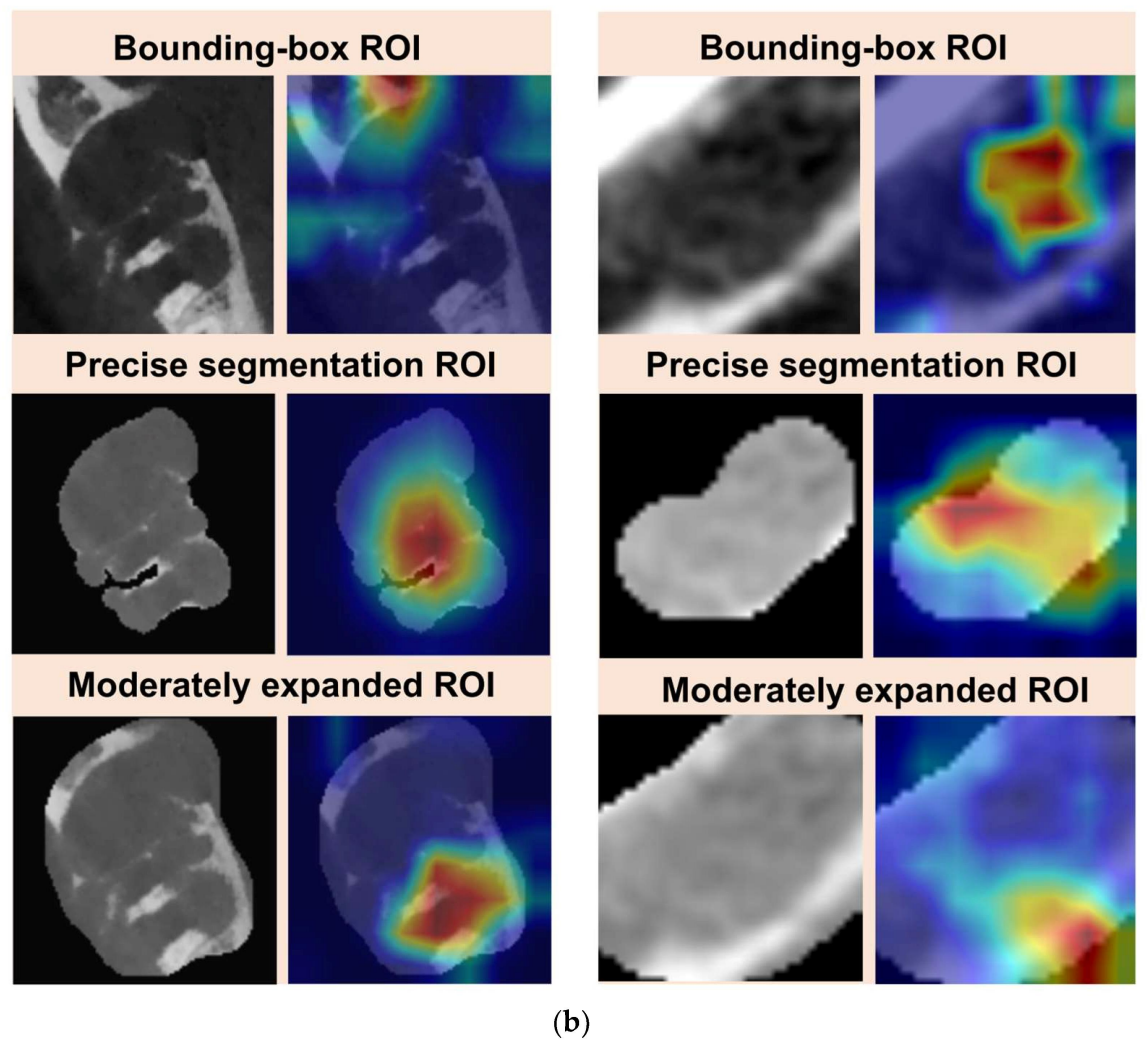
Representative attention patterns of different input types, the warmer colors indicate higher attention: (**a**) Original slice: The model’s Grad-CAM activations were dispersed over irrelevant anatomical structures. The red contour indicates the true lesion area. (**b**) Segmentation-guided ROI (Left: AME; Right: OKC): The attention pattern became structured: the bounding-box ROI led to dispersed activation; the precise segmentation ROI sharpened the focus onto the lesion; and the moderately expanded ROI shifted the focus to critical interfaces such as the septa and surrounding regions.

Figure 5a showed that the model’s attention coarsely localized to a broad region of the prominent lesion. However, more often than not, it highlighted the spine, airway, and soft tissues diverging across slices of the same lesion. Figure 5b and Figure S3 demonstrated that the segmentation-guided ROI extraction strategies influenced the model’s attention patterns. When employing a bounding-box ROI extraction strategy, the model’s attention was coarsely activated over the general region with a dispersed pattern, while the model’s attention of the precise segmentation ROI became concentrated within the lesion area and presented a tightly confined activation map. Furthermore, when a moderately expanded ROI was applied, the model’s attention exhibited a subtle shift towards the marginal zone of the lesion.

**Figure 6:**
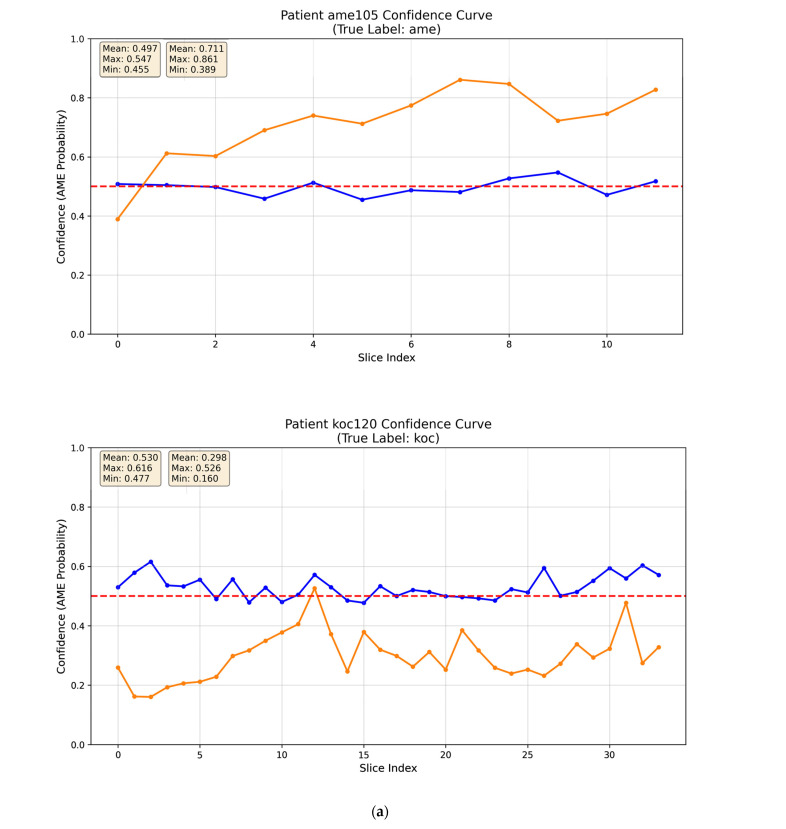

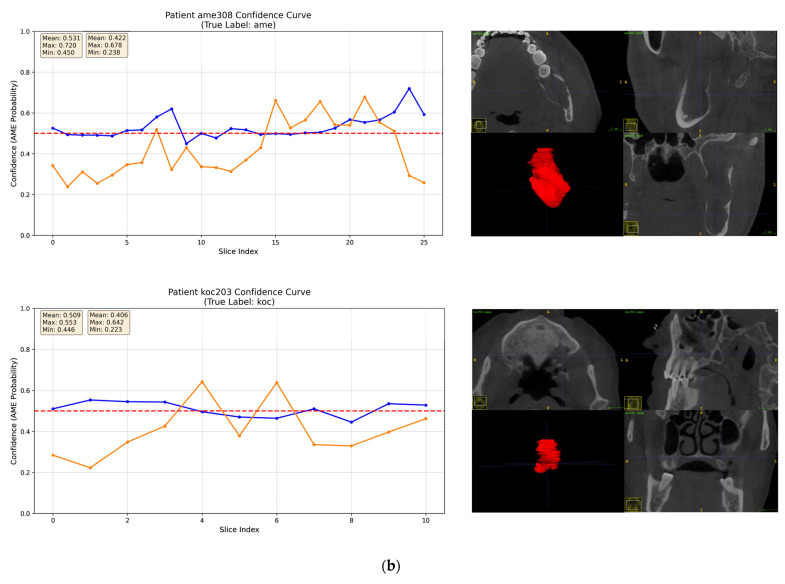
Representative confidence curve patterns: the red dashed line indicates the 0.5 decision threshold; the blue curve denotes original slices, and the orange curve denotes the moderately expanded ROI. (**a**) High-confidence pattern; (**b**) Low-confidence pattern: Case AME 308 (Unicystic Ameloblastoma) shows smooth margins and growth along the long axis, like OKC; Case OKC 203 exhibits blurred margins due to secondary infection and complex maxillary anatomy; The red area depicts their 3D lesion volumes.

Confidence curves in Figure 6 are plotted on the same axes with distinct colors for direct visual comparison. For the original slice, the confidence curves fluctuate around the 0.5 threshold in an unstructured manner, indicating that the model’s predictions were close to random chance. In contrast, the moderately expanded ROI exhibited a high-confidence pattern in most cases (Figure 6a and Figure S4), with curves that remained stable and consistently distant from the 0.5 threshold. Additionally, Figure 6b highlights two representative low-confidence cases, in which the confidence curves showed large fluctuations, reflecting uncertainty in the model’s predictions.

## 4. Discussion

This study provides a proof-of-concept that segmentation-guided ROI extraction approach is critical for deep learning-based differential diagnosis of AME and OKC using CBCT axial images. Our findings demonstrate that the moderately expanded ROI is optimal, as supported by statistically significant differences at the slice level. Although the performance difference among the three ROI strategies did not reach statistical significance at the patient level, the numerical ranking remained consistent. The lack of statistical significance at the patient level can be attributed to two main factors. First, the sample size calculations based on a target effect size of 17.7% limits the power of the DeLong’s test to detect subtle differences among already high-performing AUC. The moderately expanded strategy yielded a superior AUC (0.893) compared to the bounding-box (0.860) and precise segmentation (0.838) approaches. Second, the patient-level analysis involves probability averaging across slices, which introduces a “smoothing effect”, mitigates noisy predictions and narrows the performance gaps. Therefore, slice-level evaluation may more accurately reflect the model’s core capability for extracting discriminative diagnostic features.

Grad-CAM analysis helps explain the poor performance observed with the original slices, as the model’s attention was frequently dispersed to non-target regions. Segmentation-guided ROI extraction effectively corrected this misdirection. As shown in Figure 5b, the moderately expanded ROI enables the model to focus on prominent septa and irregular borders—key diagnostic features at the bone–lesion interface, including borders and soap-bubble structures, that differentiate AME from OKC. In contrast, the precise segmentation ROI excludes these diagnostic anchors, resulting in inferior performance. Both the bounding-box ROI and moderately expanded ROI provide the model with access to diagnostically relevant regions without requiring pixel-perfect segmentation; however, the moderately expanded ROI achieved slightly superior performance. The observed decrease in recall for the moderately expanded ROI model reflects a more conservative prediction strategy: when the model predicted AME, it was more likely to be correct, though some true AME cases may have been missed. Conversely, the bounding-box ROI model achieved the highest recall, capturing most of the true AME cases, and therefore represents a viable alternative depending on the clinical priorities of sensitivity.

Furthermore, confidence curves provide an empirical measure of the model’s diagnostic certainty. For original slices, the model often exhibited confusion, reflected in low confidence. In contrast, the moderately expanded ROI strategy produced spatially consistent and clinically interpretable prediction trajectories—high-confidence patterns—for most cases. Two representative low-confidence cases were examined in detail. Case AME308, a unicystic ameloblastoma, displayed a well-defined, smooth border and growth along the jaw axis without significant cortical expansion, resembling OKC. Case OKC203, located in the posterior maxilla, had blurred borders due to infection. In both cases, the absence of distinctive diagnostic features deprived the model of key anchors, resulting in reduced confidence. Such errors are understandable, as these cases also present diagnostic challenges for human radiologists in clinical practice.

Medical image analysis is a special scenario that requires highly interpretable diagnostics. We propose a multi-stage integration human–AI workflow that moves beyond a black-box prediction. It first automates ROI extraction to save screening time and then provides a diagnostic probability score with heatmap visualization for radiologist to review. While our current confidence curve analysis is primarily descriptive, it provides a foundation for future quantitative evaluation. For instance, the Standard Deviation (SD) of slice-level predictions offers a robust measure of diagnostic consistency. A high SD reflects a lack of spatial consensus and flags a case as a low-confidence pattern for an expert to verify. However, we emphasize that numerical metrics alone cannot fully replace visual trajectories, because identifying where a fluctuation occurs along the curve provides more actionable insights for clinicians than a single variance score. By combining Grad-CAM with the confidence curve, clinicians can check why the model is uncertain. If the heatmap focuses on artifacts or normal tissues, the clinician can disregard the AI’s output. This framework enables clinicians to assess the stability of AI predictions rather than just accept a single “black box” diagnosis.

A key methodological distinction of our study is the strict patient-centric data partition. In contrast, prior studies often split data at the slice level, which prevents the same slice from appearing in both training and test sets, yet it does not prevent different slices from the same patient from being included in both sets [6,8]. This leads to potential data leakage, as the model may learn patient-specific features during training and show overly optimistic performance. Our framework ensures that the model is evaluated on completely unseen patients and provides a more clinically valid assessment. To address the slice selection challenge, we introduce an automated method that selects slices by segmentation masks. We set an operational threshold of 50% lesion area ratio to exclude slices with very small or subtle lesions, balancing data richness with feature clarity. In addition, selecting every fifth slice is designed to balance model performance with computational resources. We believe this framework presents a practical paradigm for developing models on medical imaging modalities with variable slice counts, such as CT, MRI, and PET.

This study has several limitations. First, the high variability observed in performance metrics reflects the inherent challenge of our limited sample size and the morphological heterogeneity of jaw lesions. With only 24–26 cases per fold, the model’s performance is highly sensitive to the ‘hard’ cases. This sample size constraint, largely dictated by the labor-intensive nature of manual segmentation, also resulted in insufficient statistical significance in patient-level comparisons of three ROI strategies via DeLong’s test. Consequently, a practical next step is to develop an automated segmentation model, which is also supported by our findings that segmentation-guided preprocessing enhances downstream diagnostic accuracy and clinical confidence. By automated frameworks, future research can efficiently process larger multi-center cohorts, thereby providing the stable predictive performance required for clinical settings. The ambiguous boundaries and diverse morphologies of jaw lesions often make pixel-wise segmentation challenging. However, our findings offer a meaningful insight: moderately expanded ROIs actually outperform precise segmentations. This suggests that high-level diagnostic accuracy can be achieved without absolute pixel-wise precision. We have proposed a proof-of-concept automated framework using a Distance Vector Map (DVM) as a robust geometric descriptor for shape supervision. This approach makes reliable automated ROI extraction achievable, potentially bridging the gap toward a fully automated diagnostic pipeline. Additionally, although axial slices provide a practical starting point, their use has inherent diagnostic limitations. They poorly visualize tooth displacement and root resorption in AME and restrict broader differential diagnosis of dentigerous and radicular cysts, since the relationship of the lesion with the crown or root requires a multi-planar view. Therefore, future work will explore the integration of coronal and sagittal views using multi-view fusion (2.5D) techniques, which are less computationally demanding and require smaller sample sizes compared to full 3D analysis. Another limitation of this study is that the comparative analysis was conducted with a single Inception-V3 model. Architectures with different design principles, such as vision Transformers, might respond differently. Both the bounding-box ROI and the moderately expanded ROI capture the complete lesion and surrounding tissue, and Transformers—skilled at modeling long-range contextual dependencies—may better leverage these subtle relationships. Future studies should validate these preprocessing approaches across diverse model families to assess their robustness and architectural dependency

## 5. Conclusions

Segmentation-guided preprocessing provides an effective and accessible approach by directing model attention to diagnostically relevant regions, improving both performance and interpretability. The moderately expanded ROI proved optimal, balancing preservation of key features with background exclusion, while bounding-box ROI provides a practical alternative. Integrating Grad-CAM heatmaps with confidence curves can support a human–AI workflow, where the AI highlights uncertain cases and assists clinicians in enhancing diagnostic safety.

## Figures and Tables

**Table 1:** Confusion matrix of the proposed model for distinguishing between AME and OKC.

	Predicted OKC	Predicted AME
**True OKC**	True Negative (TN)	False Positive (FP)
**True AME**	True Negative (FN)	True Positive (TP)

**Table 2:** Comparison of Demographic and Radiographic Characteristics Between AME and OKC Groups.

Characteristic	Groups		*p*-Value
	AME (*n* = 64)	OKC (*n* = 64)	
Age (years), Median (IQR)	33 (27)	32 (27)	0.473
Sex, n (%)			
Male	32 (50)	30 (46.9)	0.724
Female	32 (50)	34 (53.1)	
Location, n (%)			
Maxilla	4 (6.3)	9 (14.1)	0.143
Mandibular	60 (93.8)	55 (85.9)	
Locularity, n (%)			
Unilocular	15 (23.4)	46 (71.9)	0.000 *
Multilocular	49 (76.6)	18 (28.1)	
Cortical Integrity, n (%)			
Intact	46 (71.9)	31 (48.4)	0.007 *
Disrupted	18 (28.1)	33 (51.6)	
Impacted Tooth, n (%)			
Present	17 (26.6)	30 (46.9)	0.017 *
Absent	47 (73.4)	34 (53.1)	
Root Resorption, n (%)			
Present	55 (85.9)	9 (14.1)	0.000 *
Absent	11 (17.2)	53 (82.8)	
CBCT Scanner, n (%)			
i-CAT FLX	50 (78.1)	49 (76.6)	0.639
New Tom 1	8 (12.5)	6 (9.4)	
New Tom 2	6 (9.4)	9 (14.1)	

Data are presented as Median (Interquartile Range, IQR) or Number (n) with Percentage (%). * *p* < 0.05.

**Table 3:** Slice-level Classification Performance Across Different ROI Extraction Strategies.

ROI Extraction Strategy	Accuracy	Precision	Recall	F1-Score
Moderately Expanded	**0.7727 ± 0.0483**	**0.8351 ± 0.0896**	0.7213 ± 0.1428	0.7603 ± 0.0761
Bounding box	0.7595 ± 0.0489	0.7630 ± 0.0384	**0.7955 ± 0.0947**	**0.7747 ± 0.0483**
Precise Segmentation	0.7309 ± 0.0604	0.7411 ± 0.0976	0.7689 ± 0.1103	0.7453 ± 0.0695
Original Slice	0.5376 ± 0.1004	0.5846 ± 0.1329	0.6583 ± 0.1519	0.5967 ± 0.0622

Note: All values represent mean ± standard deviation. Bold values indicate the best performance for each metric.

**Table 4:** Patient-level Classification Performance Across Different ROI Extraction Strategies.

ROI Extraction Strategy	Accuracy	Precision	Recall	F1-Score
Moderately Expanded	**0.8123 ± 0.0385**	**0.8649 ± 0.0950**	0.7641 ± 0.1018	**0.8009 ± 0.0441**
Bounding box	0.7975 ± 0.0744	0.7967 ± 0.0762	**0.7974 ± 0.1033**	0.7948 ± 0.0824
Precise Segmentation	0.7185 ± 0.0762	0.7155 ± 0.0947	0.7795 ± 0.1526	0.7319 ± 0.0756
Original Slice	0.4828 ± 0.1595	0.5497 ± 0.2298	0.6756 ± 0.2014	0.5686 ± 0.1101

Note: All values represent mean ± standard deviation. Bold values indicate the best performance for each metric.

## Data Availability

The datasets generated and analyzed during this study are not publicly available due to ethical restrictions and data privacy policies imposed by the participating institution to protect patient confidentiality. However, the data can be made available from the corresponding author upon reasonable request, subject to approval from the institutional ethics committee. The source code for the model implementation and analysis is publicly available at: https://github.com/zhangxinyue0417Den/CBCT-jawlesions-classification (accessed on 18 January 2026).

## Supplementary Materials

The following supporting information can be downloaded at: https://www.mdpi.com/article/10.3390/diagnostics16030416/s1, Figure S1: Bland–Altman plot and a volume correlation analysis; Figure S2: Radar charts of multi-metric performance comparison of the models at slice-level (a) and patient-level (b); Figure S3: Supplementary examples with varied cortical integrity and anatomic complexity. Left: A lesion with continuous cortical boundary. Middle: A lesion with interrupted cortical boundary. Right: A maxillary lesion with complex surrounding anatomy; Figure S4: Supplementary examples of improved diagnosis with the moderately expanded ROI; Table S1: Ablation study on slice sampling intervals: Diagnostic performance (AUC) and training efficiency; Table S2: Full model hyperparameters and computational environment; Table S3: Detailed distribution of patients and slices across 5-fold cross-validation; Table S4: Comparison of diagnostic AUCs with 95% confidence intervals.
