# Supplementary material for: Segmentation-Guided Preprocessing Improves Deep Learning Diagnostic Accuracy and Confidence of Ameloblastoma and Odontogenic Keratocyst in Cone Beam CT Images—A Preliminary Study
Source: Diagnostics (Basel). 2026 Feb 1;16(3):416. doi: 10.3390/diagnostics16030416 (PMC12897079; doi:10.3390/diagnostics16030416)
Supplement: Supplementary file 1 [file diagnostics-16-00416-s001.zip › diagnostics-3989384-supplementary.pdf]

Figure. S1 Bland-Altman plot and a volume correlation analysis

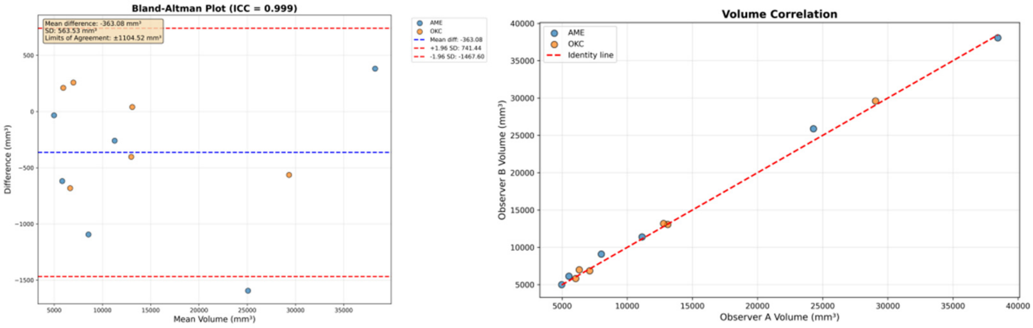

Figure. S2 Radar charts of multi-metric performance comparison of the models at slice-level(a) and patient-level(b).

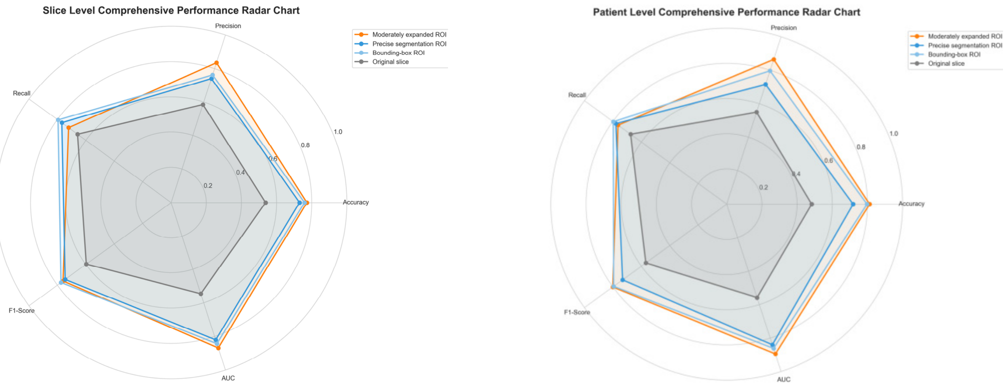

(**a**) (**b**)

Figure S3. Supplementary examples with varied cortical integrity and anatomic complexity

Left: A lesion with continuous cortical boundary.

Middle: A lesion with interrupted cortical boundary.

Right: A maxillary lesion with complex surrounding anatomy.

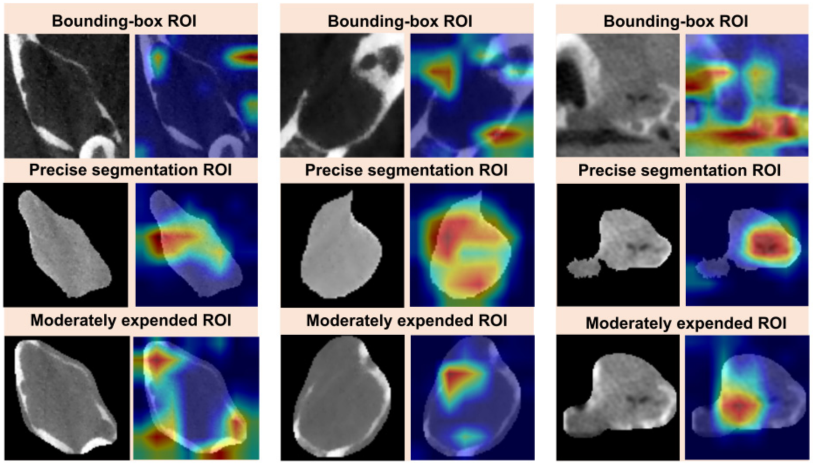

Figure S4. Supplementary examples of improved diagnosis with the moderately expanded ROI

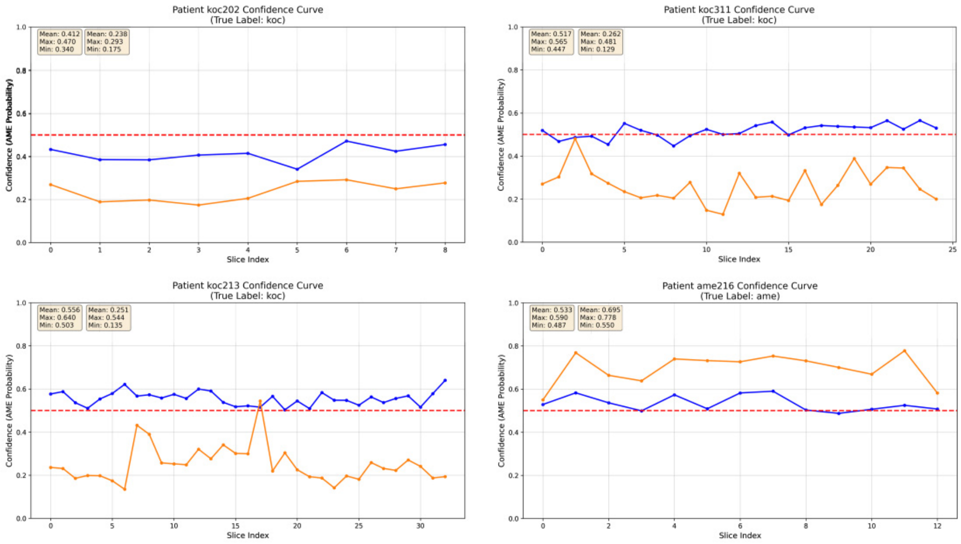

Table. S1 Ablation study on slice sampling intervals: Diagnostic performance (AUC) and training efficiency.

Sampling interval	**AUC(slice-level)**	**Time**
All slice	0.8642 ± 0.0567	10.5h
Every 3rd slice	0.871 ± 0.0437	5.5h
Every 5rd slice	0.867 ± 0.052	2h

Table. S2 Full model hyperparameters and computational environment.

**Category**	**Parameter**	**Value / Configuration**
**Optimizer**	Algorithm	AdamW
	Backbone	1e-4
	Classifier	3e-4
	Weight Decay	1e-4
**Learning Rate Scheduler**	Type	Dual-phase OneCycle Cosine Annealing
	Phase 1 (Stabilization)	Max LR: [2e-4, 6e-4]
	Phase 2 (Fine-tuning)	Max LR: [1e-4, 3e-4]
**Loss Stability**	Loss Function	Cross-Entropy
	Label Smoothing Factor	0.1
	Gradient Clipping (max_norm)	0.5
**Training Setup**	Batch Size	8 patients
	Maximum Epochs	100
	Early Stopping Patience	20
**Computational Environment**	Framework	PyTorch 2.5.1
	Programming Language	Python 3.12
	GPU	NVIDIA RTX 3090 24 GB VRAM

Table. S3. Detailed distribution of patients and slices across 5-fold cross-validation

**Fold**	**1**	**2**	**3**	**4**	**5**	**Total**
**Patients (N)**	**26**	**26**	**26**	**26**	**24**	**128**
AME/OKC	13/13	13/13	13/13	13/13	12/12	64/64
**Slices (n)**	**445**	**554**	**510**	**445**	**386**	**2,340**
AME/OKC	240/205	302/252	269/241	234/211	185/201	1230/1110

Table. S4 Comparison of diagnostic AUCs with 95% confidence intervals

ROI Extraction Strategy	AUC of slice-level		AUC of patient-level	
	**mean ± SD**	**95%CI**	**mean ± SD**	**95%CI**
**Moderately Expanded**	**0.867 ± 0.052**	**0.855-0.883**	**0.893 ± 0.049**	**0.834-0.951**
**Bounding-box**	**0.842 ± 0.045**	**0.824-0.858**	**0.86 ± 0.056**	**0.792-0.924**
**Precise Segmentation**	**0.820 ± 0.042**	**0.803-0.837**	**0.838 ± 0.059**	**0.767-0.910**
**Original Slice**	**0.545 ± 0.105**	**0.522-0.568**	**0.558 ± 0.149**	**0.459-0.658**
